# Genotypic characterization of drug resistant *Mycobacterium tuberculosis* in Quebec, 2002-2012

**DOI:** 10.1186/s12866-016-0786-4

**Published:** 2016-07-26

**Authors:** Joanna Spinato, Élyse Boivin, Émilie Bélanger-Trudelle, Huguette Fauchon, Cécile Tremblay, Hafid Soualhine

**Affiliations:** 1 McGill University, Montreal, Quebec Canada; 2 Laboratoire de santé publique du Quebec, 20045 chemin Sainte-Marie, Sainte-Anne de Bellevue, Quebec H9X 3R5 Canada; 3 Université de Montreal, Montreal, Quebec Canada; 4 Present address: Public Health Ontario Laboratory, Toronto, Ontario M5G 1 M1 Canada

**Keywords:** *M. tuberculosis*, Drug-resistance, rpoB, inhA, katG, pncA, embB

## Abstract

**Background:**

The increasing emergence of drug-resistant tuberculosis presents a threat to the effective control of tuberculosis (TB). Rapid detection of drug-resistance is more important than ever to address this scourge. The purpose of this study was to genotypically characterize the first-line antitubercular drug-resistant isolates collected over 11 years in Quebec.

**Results:**

The main mutations found in our resistant strains collection (*n* = 225) include: the S315T substitution in *katG* (50.2 %), the -15 C/T mutation in the *inhA* promoter (29 %); the S531L substitution in *rpoB* (43 %); the deletion 8 bp 446 / + R140S in *pncA* (72.9 %); the M306I (35.7 %) and M306V (21.4 %) substitutions in *embB*. Ten of the mutations in *katG* and 4 mutations identified in *pncA* were previously undescribed.

**Conclusion:**

Screening of mutations conferring resistance to first-line antituberculous drugs using DNA-sequencing approach seems to be feasible and would drastically shorten the time to determine the resistance profile compared to the proportion method.

## Background

In Canada, TB continues to affect Canadian populations disproportionately although the numbers of cases have been steadily decreasing over the past 30 years [1]. Drug resistance was found to be uncommon in Canadian-born TB patients. The highest rates of multidrug-resistant TB (MDR-TB) were from patients originating from the Eastern European region, and secondly, from the Western Pacific region. MDR-TB is defined resistance to at least isoniazid and rifampicin, whereas extensive drug resistant TB (XDR-TB) is an MDR-TB plus resistance to at least one injectable second-line drug and a fluoroquinolone. From the period of 1997 to 2008, there were 5 cases of extensively drug-resistant TB (XDR-TB), 177 cases of MDR-TB, and 1234 cases of first-line drug resistance not-MDR [2]*.* These five XDR-TB cases were reported in Ontario. The first XDR-TB case reported in the province of Quebec occurred in 2013 and concerned a patient emigrating from Eastern Europe (*H. Soualhine, pers. comm.)*

With the increase in the number of cases of drug-resistant TB steadily emerging, and more of them becoming MDR or XDR-TB, a better knowledge of the molecular mechanisms of antitubercular drug-resistance have a significant impact on the improvement of rapid detection techniques and the diagnosis of drug resistance. In Quebec, primary identification of *M. tuberculosis* complex is based on Cobas TaqMan MTB assay (Roche Diagnostics, Rotkreuz, Switzerland). Rapid detection of rifampin resistance, based on Xpert MTB/RIF assay (Cepheid, Sunnyvale, CA, USA), is performed on selected specimens. Confirmatory identification at the species level is done using a PCR-based genomic deletion analysis of RD-regions [3] and drug susceptibility testing (DST) is performed using the phenotypic MGIT 960 method (Becton Dikinson, MD, USA).

First-line antituberculous drug resistance mechanisms are well studied. Several genes have been associated with drug resistance, namely *katG* and *inhA*, with isoniazid (INH) resistance; *rpoB* with rifampicin (RMP) resistance; *embCAB* with ethambutol (EMB) resistance; and *pncA* with pyrazinamide (PZA) resistance [4–7]. Line-probe assays (LPAs) including the (INNO-LiPA Rif.TB (Innogenetics, Zwijndrecht, Belgium) and the GenoType MTBDRplus (LPA), (HainLifeScience GmbH, Nehren, Germany) and Xpert MTB/RIF (Cepheid) have been formally endorsed by the World Health Organization and are now in routine use in many TB laboratories [8]. The polymerase chain reaction sequencing-based approach is an accurate and rapid method for detection of drug-resistant TB and can identify several mutant alleles not previously associated with resistant organisms [4–7]. Recently, whole genome sequencing (WGS) of *M. tuberculosis* offers a powerful new approach to the identification of drug resistance. Furthermore, WGS of pathogens is becoming part of routine practice for establishing transmission and resistance patterns [9].

The aim of this study was to genotypically characterize resistant strains of *M. tuberculosis* circulating in Quebec, and to analyze the frequency of mutations conferring resistance to the four first-line antitubercular drugs using a DNA-sequencing approach.

## Methods

*M. tuberculosis* isolates (*n* = 2248) were obtained from clinical specimens submitted for TB culture to the Public Health Laboratory in Quebec. Resistant isolates were selected based on drug resistance profiles (*n* = 225) to the four first-line drugs: INH, RMP, PZA, and EMB. Susceptible strains (*n* = 32) were included and two reference strains were selected: *M. tuberculosis* H37Ra reference strain ATCC 25177; and *M. bovis* BCG strain ATCC 35734 that is naturally resistant to PZA.

### Drug susceptibility testing (DST)

All *M. tuberculosis* clinical isolates were tested for drug susceptibility using the BACTEC 460 instrument (Becton Dikinson, Sparks, MD, USA) from 2002 to 2005, the BACTEC MGIT 960 (Becton Dikinson) from 2010 to 2012, or by both instruments between 2006 and 2009. Critical drug concentrations used in the BACTEC 460 were INH 0.1 μg/ml, RMP 2.0 μg/ml, PZA 100 μg/ml, and EMB 2.0 μg/ml. For the BACTEC MGIT 960 instrument, critical concentrations used were INH 0.1 and 0.4 μg/ml, RMP 1.0 μg/ml, PZA 100 μg/ml, and EMB 5.0 μg/ml [10].

The drug-resistant isolates were plated on Lowenstein-Jensen slants at 37 °C. DNA was extracted using the BioRobot M48 (Qiagen, Hilden, Germany) [11]: Isolates were prepared for DNA extraction by suspending representative colonies in tubes containing beads and lysis buffer. The samples were vortexed, heated at 100 °C for 25 min, cooled on ice for 2 min, and then put in the Mini-Beadbeater (Biospec Products, Inc. Bartlesville, OK, USA) for 2 min. Finally the suspensions were centrifuged at 14 000 rcf for 2 min, before being placed in the BioRobot, and the DNA was eluted into RNase-free water.

### PCR and DNA sequencing reactions

Six pairs of oligonucleotide primers were designed to amplify regions of five genes associated with resistance to four antituberculosis drugs. PCR primers are listed in Table 1. DNA was amplified in a 50 μl mixture containing PCR buffer, 200 μM dNTP, 2 mM MgCl_2,_ 0.5 μM of each primer, 1 unit of AmpliTaq® DNA polymerase (Applied Biosystems, Carlsbad, CA, USA) and 5 μL of genomic DNA. The reaction was performed as follows: 5 min at 95 °C, followed by 40 cycles of denaturation for 30 s at 95 °C, annealing for 30 s at 60 °C, and extension for 30 s at 72 °C, with a final elongation step of 7 min at 72 °C. The amplicons were purified using a MinElute 96 UF PCR purification plates (MinElute, Qiagen) and added as template for bidirectional DNA sequencing using an ABI PRISM BigDye® terminator cycle sequencing kit v. 3.1 (Applied Biosystems, Woolston Warrington, UK). Reactions mixtures containing the forward or reverse primers were analysed with an ABI PRISM 3130xl genetic analyzer (Applied Biosystems). Bioinformatic analysis was done using the Lasergene (v. 10) software (DNAStar Inc. Madison, WI, USA). The obtained sequences were aligned in a multiple sequence alignments with the wild-type sequences of *katG*, *inhA* promoter, *rpoB*, *pncA*, and *embB*.

**Table 1:** Primers used for drug resistant gene amplification

Genes	Primers (Sequences 5’ → 3’)	Product size (bp)
*katG*	*katG-F* (5’-GACATTCGCGAGACGTTTCGG-3’)	642
	*katG-R* (5’GCTCTTAAGGCTGGCAATCTCG-3’)	
*inhA*	*inhA-F* (5’-CTATATCTCCGGTGCGGTCA-3’)	469
	*inhA-R* (5’-CTTGGCCATCGAAGCATAC-3’)	
*inhA-Prom*	*inhA-Prom-1* (5’-TCCGTCATGGTCGAAGTGTG-3’)	211
	*inhA-Prom-2* (5’-GGTAACCAGGACTGAACGGG-3’)	
*rpoB*	*rpoB-F* (5’-ACGGTCGGCGAGCTGATCC-3’)	351
	*rpoB-R* (5’-CAGACCGATGTTGGGCCCCT-3’)	
*pncA*	*pncA-F1* (5’-GGCGTCATGGACCCTATATC-3’)	670
	*pncA-R1* (5’-CAACAGTTCATCCCGGTTC-3’)	
*embB*	*embB-1* (5’- TGATATTCGGCTTCCTGCTC-3’)	417
	*embB-2* (5’- ACCGCTCGATCAGCACATAG-3’)	
∆*katG* ^a^	*katGD-1* (5’-CCGGTCAAGAAGAAGTACGG-3’)	590
	*katGD-2* (5’-CTCTTCGTCAGCTCCCACTC-3’)	
	*katGD-5* (5’-GGGGAACATCAAAGTGTCCT-3’)	400
	*katGD-6* (5’-GATACCCATGTCGAGCAGGT-3’)	

^a^Deletion of *katG*

Sensitivity and specificity were determined using the Diagnostic Effectiveness calculation from the Simple Interactive Statistical Analysis (SISA) software (Hilversum, The Netherlands) [12].

### Δ***katG*** analysis

Three INH-resistant clinical isolates repeatedly provided no amplication of *katG* gene fragment using the primer pairs previously listed. To confirm a *katG* gene deletion (Δ*katG*), amplification of other regions of this gene was investigated using different primer pairs. PCR was performed as described above with a second set of primers listed in Table 1, *katG*D-1 and *katG*D-2, designed to amplify a 590 bp fragment upstream from that amplified with the *katG* primers initially used. In addition, *katG*D-5 and *katG*D-6 were used to amplify a 400 bp region of the *katG* gene that overlaps with the region targeted by the *katG*-F and *katG*-R primers.

### Catalase-peroxidase assays

A semi-quantitative catalase assay was performed in duplicate as previously described [13], on three Δ*katG* strains and five selected INH-resistant strains with undescribed *katG* gene mutations. Controls included the reference strains of *M. tuberculosis, M. avium* ATCC 700898 for low catalase activity, and *M. gordonae* ATCC35756 for high catalase activity.

## Results

**Fig. 1:**
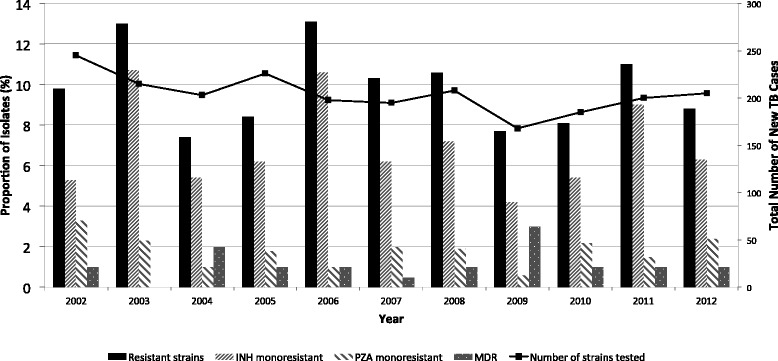
Annual profile of resistant TB in Quebec 2002-2012

The province of Quebec has reported a total of 2248 strains of *M. tuberculosis* between 2002 and 2012, of which 225 were resistant (Fig. 1). Drug resistant TB in Quebec is mainly associated with INH and PZA mono-resistance. Among the 225 phenotypically resistant isolates selected, 20 were MDR-TB. Mono-resistance patterns include 155 to INH, 3 to RMP and 41 to PZA. Six isolates were resistant to at least two first-line drugs but were not MDR. In total, 180 isolates were resistant to INH, 23 to RMP, 48 to PZA, and 14 isolates were resistant to EMB.

### *katG* gene

Out of 180 phenotypically INH-resistant isolates, 110 had a mutation in the targeted region of *katG* (61 %) (Table 2) and 3 isolates were Δ*katG*.

**Table 2:** INH-Resistance conferring mutations in 180 INH-R (85 %) and 32 INH-S (15 %) isolates

Drug resistance	*Genes*	No. of isolates	Changes	
INH^R^		180	Nucleotides	Amino acid
		3	∆ *katG*	
			Single mutation	
		1	GTC/GGC	V284G^a^
		1	AGC/AAC	S315N
		1	AGC/GGC	S315G
		78	AGC/ACC	S315T
	*katG*	1	CCG/CGG	P375R^a^
		1	TAC/TGC	Y413C^a^
		1	del G1284	frameshift^a^
		1	TGG/TCG	W438S
		1	GAC/AAC	D448N^a^
			Multiple mutations	
		1	AGC/ACC, TGA/TAA	S315T, 343Stop
		1	AGC/ACC, GAT/CAT	S315T, D387H^b^
		1	AGC/ACC, GTC/GAC	S315T, V450D^b^
		1	AGC/ACC, GGC/AGC	S315T, G451S^b^
			Single mutations	
	*inhA*	3	TCG/GCG	S94A
		1	-8 T/C	None
	*inhA-p*	37	-15C/T	None
		3	-17G/T	None
			Multiple mutations	
	*inhA-p*	1	-15C/T, -47 G/T	None
	*inhA, inhA-p*	1	GGA/GGC, -15C/T	G3G, None
		2	ATC/ACC, -15C/T	I21T, None
		3	ATC/GTC, -15C/T	I21V, None
	*katG, inhA*	1	AGC/ACC, GGA/GGC	S315T, G3G
		3	GAA/GAC, -15C/T	E261D, None
	*katG, inhA-p*	1	CCG/TCG, -15C/T	P280S^b^, None
		1	TCG/GCG, -15C/T	S303A^b^, None
		1	ACC/GCC, -15C/T	T314A, None
		1	AGC/AAC, -47G/C	S315N, None
		2	AGC/ACC, -8 T/A	S315T, None
		3	AGC/ACC, -8 T/C	S315T, None
		2	AGC/AAC, -15C/T	S315T, None
		1	AGC/AAC, -102G/A	S315T, None
		1	ACT/ATT, -15C/T	T308I, None
	*katG, inhA, inhA-p*	19	WT	None
INH^S^	*katG, inhA, inhA-p*	32	WT	None

^a^Undescribed mutations that occur alone; ^b^undescribed mutations that occur in combination with another resistance-associated mutation; inhA-p, inhA promoter, del, deletion; *WT* wild-type sequence, *R* resistant, *S* susceptible

A total of 10 mutations in *katG* gene were novel and found in INH monoresistant strains; five undescribed substitutions occurred alone (V284G; P375R; Y413C; del G1284 and D448N) and five substitutions occurred in combination with a mutation known to confer INH-resistance, among them P280S and S303A occurred with -15 C/T in *inhA* promoter while D387H; V450S; and G451S occurred with S315T in *katG*. The main mutation in *katG* (50.2 %) was the AGC/ACC (S315T). In 8.8 % of the S315T mutants, *inhA* promoter mutations were also identified. Three INH-resistant isolates demonstrated no amplification of the *katG* gene fragment using the primer pairs katG-F/katG-R, katGD-1/katGD-2 and katGD-5/katGD-6. No catalase activity was observed by the semi-quantitative catalase assay on those three clinical isolates compared to the control strains of *M. tuberculosis*, the weak catalase producer *M. avium* and the strong producer, *M. gordonae,* which showed an average of 28 mm, 9 mm and 57 mm of oxygen bubble production respectively. The *katG* mutated strains (V284G; P375R; Y413C; del G1284 and D448N) identified in our study, have shown a catalase activity of 19 mm, 21 mm, 38 mm, 11 mm and 27 mm respectively.

### *inhA* gene

Eight out of the 180 INH-resistant isolates (4.4 %) contained a single point mutation in the *inhA* gene. Three isolates had an I21V substitution, 2 had an I21T substitution, and 3 had a S94A substitution. The substitutions at codon 21 always occurred in combination with the -15 C/T mutation in the *inhA* promoter.

### *inhA* promoter

Isolates with a mutation in the *inhA* promoter made up a total of 36 % of INH-resistances. The most commonly encountered mutation was -15 C/T (29 %), followed by mutations at position -8 (3.9 %). The mutations at -47 and -102 always occurred in combination with the -15 C/T inhA-promoter mutation, the S315T, or the S315N substitution in *katG*, which are mutations known to confer INH-resistance.

### *rpoB*

All RMP-resistant isolates contained a mutation in the targeted region of the *rpoB* gene (Table 3): One had an insertion of F514 (TTC), 18 had a single point mutation, and 4 contained two point mutations. All mutations occurred within the RMP resistance determining region (RRDR) except T480I (ACC/ATG); this mutation occurred in combination with the S531L substitution. The most common mutation observed in *rpoB* was at codon 531, such as the S351L substitution, which was found in 47.8 % of the isolates. Substitutions at codon 526 were found in 26 % of isolates. Two L511R substitutions occurred in combination with D516Y.

**Table 3:** Characteristic of mutations found in resistant isolates to RMP (*n* = 23), PZA (*n* = 48) or EMB (*n* = 14) compared to susceptible strains (*n* = 32)

Drug resistance	*Genes*	No. of isolates	Changes	
RMP^R^	*rpoB*	23	Nucleotides	Amino acid
			Single mutations	
		1	TTC514	Ins F514
		2	GAC/GTC	D516V
		4	CAC/GAC	H526D
		2	CAC/CGC	H526R
		8	TCG/TTG	S531L
		1	TCG/TTT	S531F
		1	CTG/CCG	L533P
			Multiple mutations	
		2	CTG/CGG, GAC/TAC	L511R, D516Y
		2	TCG/TTG, ACC/ATG	S531L, T480I
RMP^S^	*rpoB*	32	WT	None
PZA^R^	*pncA*	48		
			Single mutations	
		1	-11A/G	None
		1	CTG/CCG	L27P
		1	Del G164	Frameshift^a^
		2	ATC/AGC	I90S
		1	GCG/CCG	A92P^a^
		1	Del 9 bp 380	Del D,E,V^a^
		1	ACC/CCC	T135P
		1	Ins AT 447	Frameshift^a^
		1	ACC/CCC	T168P
			Multiple mutations	
		35	Del8 bp446, CGC/AGC	Frameshift, R140S
		1	GCA/GCG	A46A
		2	WT	None
PZA^S^	*pncA*	32	WT	None
EMB^R^	*embB*	14		
			Single mutations	
		4	ATG/ATA	M306I
		3	ATG/GTG	M306V
		2	TAT/TCT	Y319S
		1	GAT/TAT	D328Y
		1	GAC/GCC	D354A
		1	GGC/AGC	G406S
			Multiple mutations	
		1	ATG/ATA, CTG/CTA, GAG/GCG	M306I, L355L, E387A
		1	WT	None
EMB^S^	*embB*	32	WT	None

^a^Undescribed mutations; *Del* deletion, *Ins* insertion, *WT* wild-type sequence, *R* resistant, *S* susceptible

### *pncA*

Forty-five out of 48 PZA-resistant isolates had a mutation in the *pncA* gene or in the *pncA* promoter (93.7 %). As shown in Table 3, 35 PZA-monoresistant isolates had a mutation, identified only in Quebec, which consist of a deletion of 8 bp at position 446 (frameshift) in combination with the R140S substitution (CGC/AGC). Two PZA-resistant strains harboured no mutation in the *pncA* gene and one PZA-resistant strain contained only a silent mutation, A46A (GCA/GCG).

### *embB*

Thirteen of 14 EMB-resistant isolates had a mutation in the *embB* gene (92.8 %). The most commonly found substitution occurred at codon 306 (57 %). Other substitutions include Y319S, D328Y, D354A, G406S, and E378A. The isolate with the E378A substitution also had a substitution in codon 306 and a silent mutation in codon 355.

### Mutations and MDR-TB

Twenty isolates were MDR-TB as defined by resistance to both INH and RMP. The most common mutation combination seen in the MDR-TB isolates was in *rpoB*531 and *katG*315 (40 %), followed by *rpoB*526 and *katG*315 (15 %), and *inhA* promoter -15 with *rpoB*531 (15 %) among others (Table 4).

**Table 4:** Characteristic of mutations found in 20 MDR isolates

No. of MDR isolates	RMP		INH		EMB	PZA
	*rpoB*	*katG*	*inhA*	*inhA-p*	*embB*	*pncA*
1	L511R, D516Y	S315T				
1	L511R, D516Y	S315T				del 9 bp 380 (del D,E,V)
1	Ins F514	S315T		T-8A		T168P
1	D516V	S315T		T-8A	G406S	InsAT 447
1	D516V	E261D		C-15 T		
1	H526D	S315T				
1	H526D	S315T			M306I	
1	H526D	S315T			D328Y	
1	S531F	S315T				
2	S531L	S315T				
1	S531L	S315T		C-15 T	Y319S	
2	S531L			C-15 T		
1	S531L		I21T	C-15 T	M306I	
1	S531L	S315T			D354A	T135P
1	S531L	S315T		T-8C		A-11G
2	S531L, T480I	S315T			M306V	
1	L533P	S315T			M306I	L27P

*inhA-p* inhA promoter, *del* deletion, *Ins* insertion

## Discussion

Phenotypic DST is performed using the BACTEC MGIT 960 system. While this method is termed the gold standard for determining drug susceptibility profiles of *M. tuberculosis* isolates, it can take two weeks before results are available. Limitations of phenotypic methods include the uncertain reliability of conventional breakpoints, decreased accuracy in cultures mixed with other mycobacteria, and the possibility of reduced fitness and growth of mutant organisms, which may require a higher inoculum to increase test sensitivity. Further, DST to PZA can be hampered by false-positive results [14].

In our study, DNA sequencing is applicable for the detection of INH-resistant isolates with 60.8 % sensitivity (95 % CI, 51.7 to 69.8) and 100 % specificity for the *katG* gene and with 35.9 % sensitivity (95 % CI, 27 to 44.8) and 100 % specificity for the *inhA* promoter. Combined the two tests have 85.1 % sensitivity (95 % CI, 78.5 to 91.7) and 100 % specificity.

Ten mutations identified in the *katG* gene of the INH-resistant isolates were undescribed according to our knowledge. Five substitutions occurred in combination with resistance-associated mutations, meaning that the resistance observed with these isolates can be attributed to the well-established resistance-conferring mutations; these undescribed mutations could also be associated with fitness cost of INH resistance [15]. The remaining five undescribed substitutions that occurred alone (V284G, P375R, Y413C, del G 1284, and D448N) require further analysis. Five strains harboring these mutations showed catalase activity. Among them, 3 strains (V284G, P375R, and del G 1284) showed a reduced catalase activity compared to the control strains.

In our isolates, the S315T substitution accounted for 50.2 % of the *katG* gene mutations. This particular substitution has also been reported to occur more frequently in MDR-TB isolates, presumably because it provides INH resistance with the retention of virulence [16]. This would allow the S315T mutated strains to persist in the intracellular environment and possibly become resistant to additional antitubercular drugs. In three strains of our collection, the deletion of *katG* resulted in no KatG enzymatic activity, and therefore, INH cannot be converted into its active form. It was shown that when KatG activity has been completely lost, *ahpC* promoter mutations are usually found. The *ahpC* gene encodes an enzyme involved in antioxidant defense, as does the *katG* gene product [13]*.*

*inhA* promoter mutations made up 36 % of the INH-resistant isolates. Two rarely reported mutations were also identified at positions -47 and -102. As these two mutations always occur in combination with the S315T substitution in *katG* as previously found, their consequences are unknown at the present time.

In contrast, there were 19 INH-resistant isolates that did not have any mutations in *katG, inhA,* or the *inhA* promoter; other mechanisms of resistance could be at play. Mutations in other genes such as *ahpC*, *kasA*, *ndh,*and *mshA* have also been reported [17], in addition to efflux-related mechanisms [18, 19].

We have identified *rpoB* mutations in 100 % of the RMP-resistant isolates. Previous studies indicate that mutations at codons 531, 526, and 516 make up the majority of RMP-resistance mutations reported [20]. All mutations identified in the *rpoB* gene occurred within the RRDR except one substitution, T480I, which occurred outside of this core region. Both isolates that had the T480I substitution also had S531L substitution; it is unclear whether the T480I substitution is involved in RMP resistance [21]. One insertion of F514 was identified in our collection. This insertion was also reported in a strain isolated from the same patient in Ontario [22].

RMP mono-resistance is rarely reported [20]. In this study 87 % of the RMP-resistant isolates were also resistant to INH, making them MDR-TB. The molecular tests could therefore be used to identify the majority of MDR-TB cases. The automated PCR test utilizing the GeneXpert platform, which can accurately detect TB as well as RMP resistance within 2 h, is useful for detecting MDR-TB [8]. As with the line probe assay, not all mutations will be identified in the *rpoB* gene that confers RMP resistance. Novel mutations cannot be identified, and other mutations cannot be detected with these assays [23–25].

Mutations in the *pncA* gene and promoter accounted for 93.8 % of PZA resistance. DNA sequencing had detected PZA resistance with a sensitivity of 93.8 % (95 % CI, 85 to 100) and specificity of 100 %. Among the Canadian-born population in Quebec, a high proportion of TB isolates are monopyrazinamide-resistant comparing to the other provinces [2, 26]. In the absence of a recognized outbreak, it was hypothesized that these isolates most likely represented reactivation of an old endemic strain [26]. The most common mutation seen in the province was a deletion of 8 bp at position 446 combined with a R140S substitution (72.9 %). Previous studies have shown that strains carrying this mutation are related [26, 27] and suggested that the clonal PZA-resistant strains carrying this mutation are actively being transmitted, rather than acquired resistance individually in the patient via the selective pressures of drug exposure [26].

Four novel mutations found in our collection consisted of a deletion (G at position 164) in INH and PZA-resistant strain; an insertion (AT at position 447) and a deletion of residues D, E, and V at position 380 found in 2 MDR strains, and the A92P substitution in one monoresistant strain. There were 3 PZA-resistant isolates that did not have a mutation in the *pncA* gene; this cannot rule out a false resistant result by the phenotypic method [14]. Furthermore, not all PZA-resistant *M. tuberculosis* isolates have mutations in this gene [28], which is suggestive of other mechanisms of resistance. For instance, mutations in the *rpsA* gene, encoding ribosomal protein S1, have been described as a mechanism for PZA resistance. A silent mutation of A46A was identified in one isolate. According to Somoskovi et al., this SNP is reported to serve as a marker of *M. canettii*, which is naturally resistant to PZA, but not as a result of *pncA* mutations [29]. This strain, which was probably misidentified as *M. tuberculosis*, since it also contains the RD9 region, showed smooth colonies, characteristic of *M. canettii* [3]. Other mechanisms reported to cause PZA resistance in *M. tuberculosis*, *M. canettii*, and *M. bovis* include: altered PZA uptake and/or pyrazinoic acid efflux [30].

EMB-resistance conferring mutations were identified in 13 out of 14 EMB-resistant isolates (sensitivity of 92.8 % (95 % CI, 75.6 to 100) and a specificity of 100 %). Fifty-seven percent of identified substitutions occurred at codon 306 which is the most common site reported for changes associated with EMB resistance [31]. A substitution, E378A, was found in one EMB-resistant clinical isolate in our collection. The same mutation was also identified in one EMB-susceptible clinical isolate, and in the EMB-susceptible control, *M. bovis* BCG TMC1011 as was previously reported upon whole genome sequencing [32]. Our EMB-resistant isolate also contained the M306I substitution. The substitution caused by E378A in the BCG and clinical isolate are not associated with EMB-resistance, and are reported as polymorphism that is not resistance related.

One EMB-resistant isolate did not have mutations in the *embB* gene, which may indicate other mechanisms of EMB resistance do exist [33]. An efflux pump mechanism has been associated with resistance to EMB [18], and occasionally resistance-conferring mutations have also been reported in *embC* [34].

## Conclusion

DNA sequencing was able to detect mutations in the targeted genes: *katG* (62 %); *inhA* promoter (36 %); *inhA* gene (4.4 %); *katG* and *inhA* promoter together (89.5 %). From RMP, PZA, and EMB, 100 %, 93.7 %, and 92.8 % of resistant strains showed a mutation in *rpoB*, *pncA*, and *embB*, respectively. Comparing to the phenotypic methd, DNA sequencing of targeting genomic regions associated with drug resistance would drastically shorten time to resistance profile determination in clinical isolates collected in the region.

It is of great importance to note that the absence of a mutation does not imply drug susceptibility; therefore, it is essential that molecular methods must always be compared to the gold standard method.

## Abbreviations

CI, Confidence Interval; DST, drug susceptibility testing; EMB, ethambutol; INH, isoniazid; MDR-TB, multidrug-resistant tuberculosis; PZA, pyrazinamide; RMP, rifampin; RRDR, rifampin resistance determining region; SISA, simple interactive statistical analysis; XDR-TB, extensively drug-resistant tuberculosis; ΔkatG, katG gene deletion.
